# The role of environmental factors on sporadic Creutzfeldt-Jakob disease mortality: evidence from an age-period-cohort analysis

**DOI:** 10.1007/s10654-023-01004-5

**Published:** 2023-05-16

**Authors:** Angéline Denouel, Jean-Philippe Brandel, Danielle Seilhean, Jean-Louis Laplanche, Alexis Elbaz, Stéphane Haik

**Affiliations:** 1 CNRS UMR 7225, INSERM U1127, Paris Brain Institute, Sorbonne Universités, Paris, France; 2 AP-HP, Centre National de Référence des Maladies de Creutzfeldt-Jakob, Groupe Hospitalier Pitié-Salpêtrière, Paris, France; 3 Département de Biochimie et Biologie Moléculaire, Hôpitaux Lariboisière-Fernand Widal, Paris, France; 4 INSERM, UMR 1144, “Optimisation Thérapeutique en Neuropsychopharmacologie”, Paris, France; 5 Université Paris-Saclay, UVSQ, Univ. Paris-Sud, Gustave Roussy, Inserm, U1018, Team « Exposome, Heredity, Cancer, and Health », CESP, Villejuif, 94807 France

**Keywords:** Age-period-cohort model, Prion, Temporal trend, Sporadic Creutzfeldt-Jakob disease

## Abstract

Sporadic Creutzfeldt-Jakob disease (sCJD) is the most common form of prion diseases. The causes of sCJD are still unknown and exogenous factors may play a role. Worldwide, the number of patients with sCJD has progressively increased over time. This increase can be partly explained by increasing life expectancy and better case ascertainment, but a true increase in the number of sCJD cases cannot be excluded. We estimated mortality rates from sCJD in France (1992–2016) and studied variation in mortality rates by age, period, and time.

We included all cases aged 45–89 years old who died with a probable/definite sCJD diagnosis based on the French national surveillance network. We used age-period-cohort (APC) Poisson regression models to study variation in mortality rates by sex, age, period, and time.

A total of 2475 sCJD cases aged 45–89 years were included. Mortality rates increased with age, reached a peak between 75 and 79 years, and decreased thereafter. Mortality rates were higher in women than men at younger ages and lower at older ages. The full APC model with a sex×age interaction provided the best fit to the data, thus in favour of sex, age, period, and cohort effects on mortality rates. In particular, mortality rates increased progressively with successive birth cohorts.

Based on 25 years of active surveillance in France, we show evidence for sex, age, period, and cohort effects on sCJD mortality. The identification of cohort effects suggests that environmental exposures may play a role in sCJD etiology.

**Supplementary Information:**

The online version contains supplementary material available at 10.1007/s10654-023-01004-5.

## Introduction

Transmissible spongiform encephalopathies (TSE) are rare transmissible neurodegenerative disorders caused by the brain accumulation of a misfolded, partially protease-resistant isoform (PrP^Sc^) of the host-encoded cellular prion protein PrP^c^ [1, 2]. In humans, prion diseases are observed in different forms, the most common being sporadic Creutzfeldt-Jakob disease (sCJD). Different sCJD subtypes exist that are associated with different human prion strains [3]. The etiology of the other forms is well known, including: mutations in the gene encoding PrP for inherited prion diseases; oral exposure to the agent responsible for classical bovine spongiform encephalopathy (BSE) in variant Creutzfeldt-Jakob disease form (vCJD); use of contaminated instruments or materials from human origin such as growth hormone extracted from cadaver-sourced pituitary glands or dura mater graft in iatrogenic forms (iCJD). Alternatively, the origin of sCJD remains unclear and different hypotheses have been proposed. Sporadic forms could occur endogenously as the result of random somatic mutations within the CNS or of a spontaneous conversion of PrP^c^ into PrP^Sc^ [2]. Another hypothesis is based on an exogenous origin of sCJD. Several case-control studies attempted to identify potential risk factors, including animal exposure, diet, professional exposures, and medical or surgical history [4–11], but failed to deliver consistent results, possibly due to potential biases or study design issues, such as source of controls, exposure assessment, or recall bias [12].

Worldwide, the number of patients with sCJD appears to have progressively increased over time [13]. This increase can be partly explained by increasing life expectancy as well as by better case ascertainment due to improved diagnostic tests and awareness of the disease among clinicians. Indeed, a relationship between surveillance intensity and sCJD incidence has been shown [14]. It cannot be excluded, however, that an actual increase of sCJD cases has occurred, and this hypothesis can be examined using age-period-cohort (APC) models.

The APC model is a descriptive modeling tool that allows describing rates as a product of age, period, and cohort effects, and assessing variation by age and time trends in the rates [15]. Age effects are considered to reflect biologic processes related to aging. Period effects, which affect all age groups at a given time, reflect a global trend related, for example, to changes in diagnosis, case identification, or treatment. Cohort effects, seen as a change in the rates for a cohort of individuals born during the same period of time, can be due to changing environmental exposures. The APC approach requires large sample sizes that have been difficult to achieve for such a rare disease and, to our knowledge, it has been used only once based on 716 patients identified in the United Kingdom (1970–1997) [16]. This study, however, considered period and cohort effects independently of one another, and did not perform a full APC analysis.

In this paper, we estimated mortality rates from sCJD in France over a 25-year period (1992–2016) based on data from the French national surveillance network. We examined sex-differences and used an APC approach to study variation in mortality rates by age, period, and time. Analyses were first performed overall and then restricted to the most frequent prion strain.

## Materials and methods

### National CJD surveillance network in France

All patients identified by the French CJD surveillance Network who died over a 25-year period (01/1992-12/2016) with a probable or definite sCJD diagnosis were included in the analyses. Possible sCJD cases were excluded. Additional details on data collection and molecular classification of subtypes/strains are provided in supplementary (eMethods).

### Statistical analysis

Analyses were performed using Stata/SE 16.1 (StataCorp, College Station, TX). The *apcfit* package was used to implement the APC model [17]. We used a two-tailed significance threshold of 0.05.

#### Mortality rates

Mortality rates (per 1,000,000 person-years) were computed as the ratio of the observed number of deaths to the person-years at risk from the French population, overall and by sex and 5-years age groups; confidence intervals (CI) were computed using Poisson regression. We modeled the non-linear relation between mortality rates and age through a Poisson regression model with continuous age (defined by the midpoints of each age group) as linear, quadratic and cubic terms.

To examine temporal trends in mortality, we computed age- and sex- standardized mortality rates each year between 1992 and 2016 using the French population (1992–2016) as the reference.

#### Male-to-female mortality ratios

To examine sex-differences, we computed age-standardized mortality rates in men and women, overall and by year, using direct standardization with the French population (1992–2016) as the reference.

We estimated age-specific male-to-female (M/F) mortality ratios by dividing mortality rates in men by rates in women for each 5-years age group; 95% CI and p-values were computed using Poisson regression. The age-by-sex interaction was tested by including a multiplicative term between age groups and sex. We then modeled the non-linear relation between M/F ratios and continuous age through a Poisson regression model including sex, continuous age as linear, quadratic and cubic terms, and their interaction with sex.

#### Age-period-cohort model

In order to study time trends in sCJD mortality rates, we used an APC model with three time variables categorized in 5-years intervals: age at death (45–89 years), period of death (1992–2016), cohort of birth (1907–1967).

We first plotted sCJD mortality rates by age groups for different periods, by period for different age groups, by age groups for different cohorts of birth, and by cohort of birth for different age groups [15]; given sex-differences in the analyses described above, these plots were stratified by sex.

We then implemented the APC model using the approach described by Carstensen [15]. This method aims at solving the identifiability issue of APC models (due to the linear relationship between the three variables: cohort = period–age), by modelling the variables as spline functions. We used the midpoint of age and period categories and restricted cubic splines with 5 degrees of freedom for age and cohort and 3 degrees of freedom for period. We considered the following models: age + period (AP), age + cohort (AC), age + period + cohort (APC). Deviance divided by the degrees of freedom and the Akaike’s information criterion (AIC; lower values indicate better fit) were used to assess models’ fit. We compared nested models using likelihood ratio tests.

The full APC model can be parametrized in different ways. Previous European studies reported increasing sCJD mortality rates in the late 1990s’ in countries in which prospective surveillance systems had been set up; increasing rates were interpreted as reflecting better disease ascertainment, thus in favour of period effects. Our primary interest was therefore to determine whether cohort effects also exist; accordingly, we used an APC parametrization according to which age effects represent rates for the reference cohort, cohort effects are expressed as relative risks (RR) relative to the reference cohort (1937), and period effects are expressed as RRs constrained to be 0 on average (on the log scale) with 0 slope. The period function represents a residual relative risk from the rate predicted by age-cohort combination.

Based on our finding of sex-differences in mortality rates of sCJD, we then included sex in the models and examined interactions of sex with age, period, and cohort [17]. We started by fitting a full model including all multiplicative interactions between sex and splines for the three time variables, and simplified this model by sequentially excluding non-significant interactions one at a time.

In sensitivity analyses, in order to describe period effects, we used an alternative parametrization according to which age effects represent rates for the reference period, period effects are expressed as relative risks (RR) relative to the reference period (2007), and cohort effects are expressed as RRs constrained to be 0 on average (on the log scale) with 0 slope. The cohort function represents a residual relative risk from the rate predicted by age-period-combination.

Classically, APC models were first implemented by fitting age, period and cohort effects as factor variables; this approach was used in the only other available study performed in the United Kingdom, although it only considered AP and AC models [16]. We used this method in sensitivity analyses, and we compared the results of the analyses described above to this approach. One disadvantage of this approach is that, in order to solve the identifiability problem, an arbitrary constraint on at least one parameter is necessary [18]. We fixed the regression coefficients corresponding to two periods of death (2002–2006, 2007–2011) to be equal. This choice was based on the hypothesis that the initial improvement of case identification over the first years of the network stabilized after 2002, in agreement with the results of our main analysis. We used a stepwise procedure by building models of increasing complexity: Age (A), age + drift (AD, where drift represents a constant linear effect that is not attributable to period or cohort effects), AP, AC, APC. We used the same approach described above to assess the models’ fit and to compare nested models.

These analyses were first performed for all sCJD cases. We then restricted our analyses to sCJD cases with the M1 strain in order to examine whether the pattern detected overall was confirmed for the most frequent strain.

### Data availability

All the results from the paper can be reproduced using the figures provided in the tables.

## Results

### Mortality rates

Between 1992 and 2016, 2,510 cases died with a probable or definite sCJD diagnosis. Analyses are restricted to 2,475 patients (1,097 men, 1,378 women) aged 45–89 years old at the time of death, because there were few younger and older patients (25 cases < 45 years; 10 cases ≥ 90 years).

**Fig. 1:**
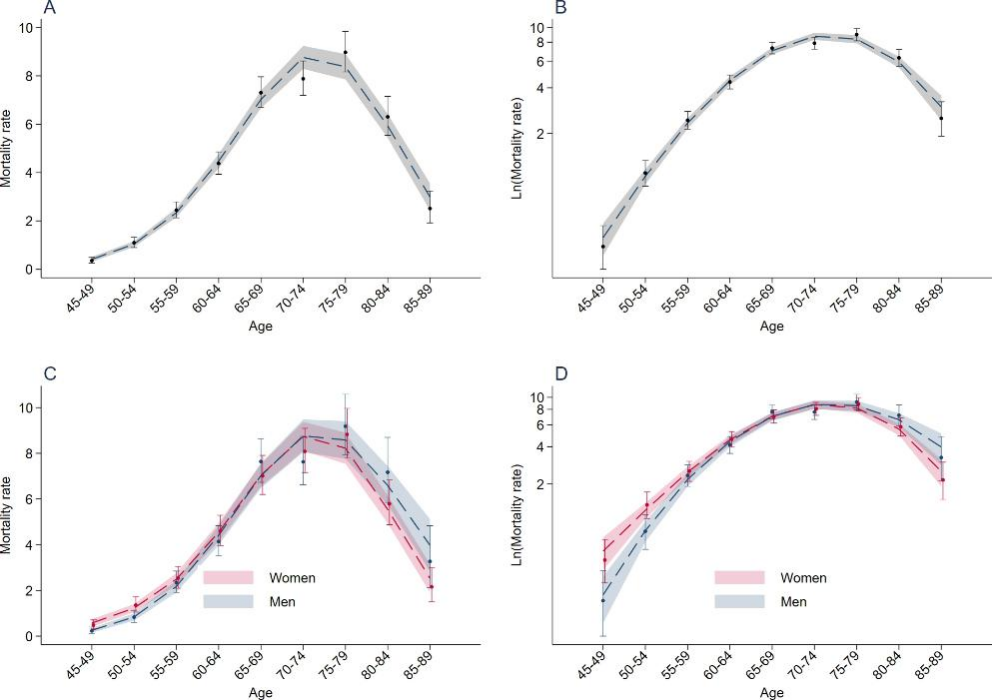
Age-specific mortality rates of sCJD (France, 1992–2016). Circles (point estimates) and vertical bars (95% confidence intervals) correspond to age-specific observed mortality rates of sCJD (per 1,000,000 person-years), overall and by sex (corresponding numbers are shown in Table S1). Figures B and D use a logarithmic scale for the Y-axis. The dashed line corresponds to mortality rates predicted by Poisson regression including age, age², and age^3^ (shaded area, 95% confidence intervals; Table S2 shows the regression coefficients from the model)

The overall sCJD mortality rate was 4.58 per 1,000,000 person-years (95% CI = 4.39–4.78) (Table S1). Overall and in both sexes, mortality rates increased with age, reached a peak between 75 and 79 years, and decreased thereafter. Figures 1A and 1B show a good fit between observed mortality rates and those predicted by Poisson regression including age, age^2^, and age^3^ (regression coefficients shown in Table S2).

Standardized mortality rates rapidly increased over the first years of the surveillance network from 0.73 (95% CI = 0.727–0.728) in 1992 to 1.47 (95% CI = 1.467–1.469) per 1,000,000 person-years in 1997 (Table S3 & Figure S1). Standardized mortality rates continued to slowly increase over subsequent years with yearly fluctuations.

### Male-to-female mortality ratios

The overall crude mortality rate for patients aged 45–89 years was higher in women (4.15 per 1,000,000) than men (3.83 per 1,000,000). Age-standardized mortality rates were 4.54 per 1,000,000 person-years (95% CI = 4.29–4.79) in women and 4.72 per 1,000,000 person-years (95% CI = 4.40–5.03) in men.

**Fig. 2:**
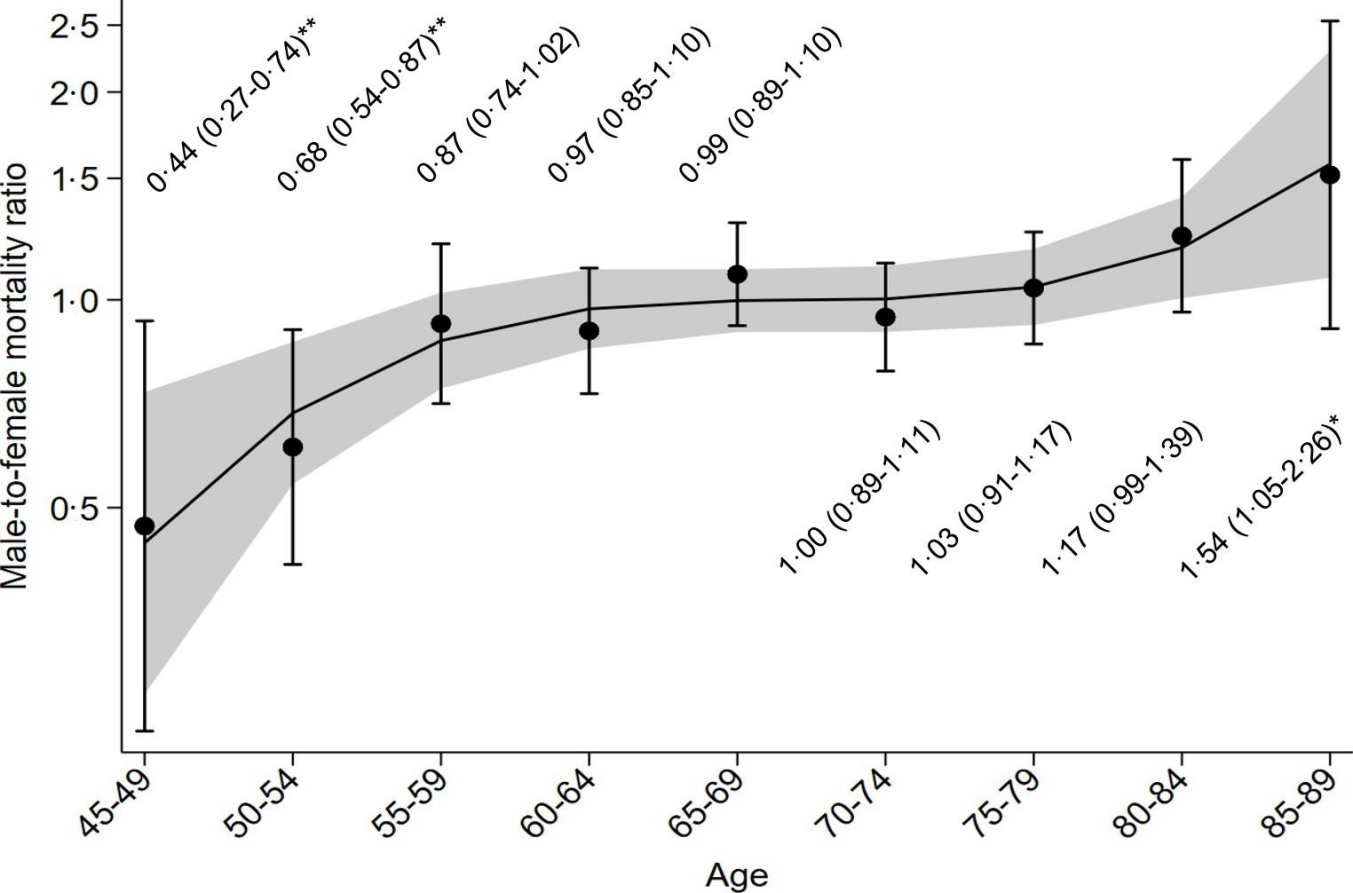
Non-linear relation between male-to-female mortality ratios of sCJD (France, 1992–2016) and age. Circles (point estimates) and vertical bars (95% confidence intervals) correspond to age-specific observed male-to-female (M/F) mortality ratios of sCJD. The black line corresponds to M/F ratios predicted by Poisson regression including age, age^2^, and age^3^ (shaded area, 95% confidence intervals; Table S2). *P < 0.05. **P < 0.01

Age-specific M/F mortality ratios increased progressively as age increased (interaction between age groups and sex: *P* = 0.002; Figure 2). Mortality rates were higher in women than men between 45 and 49 (M/F = 0.44, *P* = 0.002) and 50–54 years (M/F = 0.68, *P* = 0.002), while they were higher in men than women between 80 and 84 (M/F = 1.17, *P* = 0.067) and 85–89 years (M/F = 1.54, *P* = 0.027).

Figures 1C and 1D show that there was a good fit between observed mortality rates and those predicted by Poisson regression including an interaction between sex and age, age^2^, and age^3^ (regression coefficients shown in Table S2). Figure 2 shows the cubic relation between M/F mortality ratios and age according to this model.

### Age-period-cohort model

Mortality rates tabulated by sex, age and period are shown in Table S4. Plots of mortality rates of sCJD by age at death, period of death, and cohort of birth are presented for men and women separately showing similar patterns in both sexes (Figure S2). Mortality rates increased with period in all age groups, with the largest increase noted between 1992 and 1996 and smaller differences across subsequent periods (Figures S2A and S2B). At all ages, mortality rates increased with cohort of birth, with older cohorts showing lower mortality rates (Figures S2C and S2D).

**Fig. 3:**
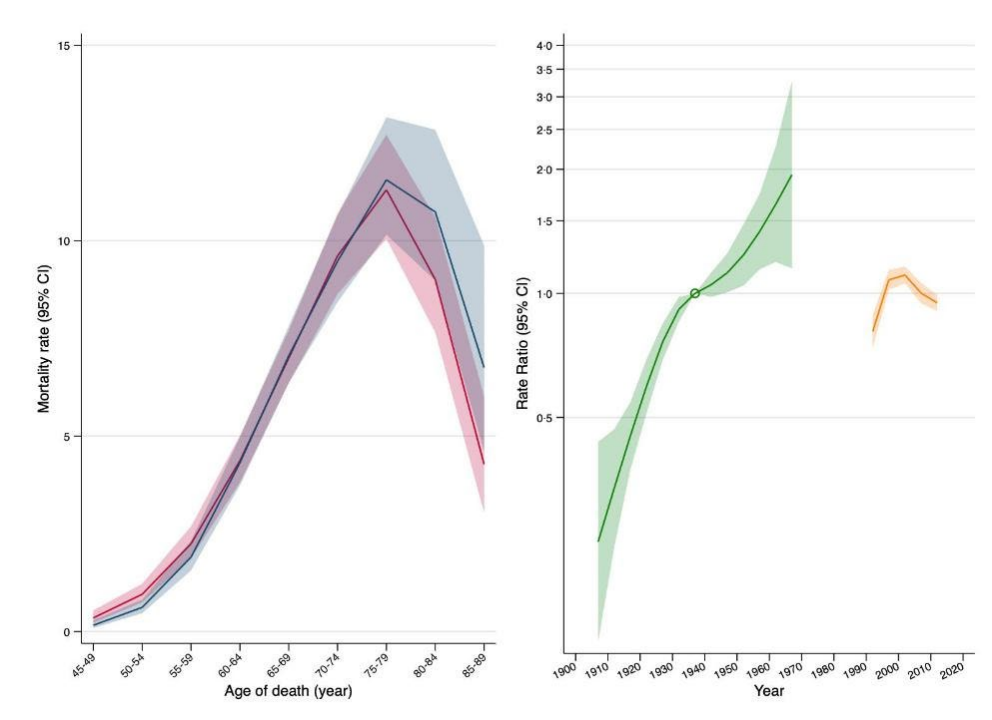
Age, period, and cohort effects for sCJD mortality rates. The left part of the graph shows sCJD age-specific mortality rates (per 1,000,000 person-years; shaded area, 95% confidence intervals) in men (blue) and women (red). The right part of the graph shows relative risks (green; shaded area, 95% confidence intervals) for cohorts (reference cohort, 1937), and average period effects (orange) constrained to be zero on average

Table 1 shows the results of APC models. The full APC model had the smallest AIC and provided a significantly better fit to the data than the AP (*P* = 0.002) and AC (*P* < 0.001) models. We then added sex and its interaction with the three time variables. The sex×period (*P* = 0.898) and sex×cohort (*P* = 0.814) interactions were not statistically significant and were deleted. We retained the sex×age interaction that was statistically significant (*P* = 0.007); the model including this interaction had a lower AIC and provided a better fit than the model without this interaction (*P* = 0.012). Predictions of mortality rates by age, period, and cohort according to this model are shown in Figure 3. Mortality rates were higher in women than men at younger ages, but became higher in men than women at older ages. Mortality rates increased progressively with successive birth cohorts. For instance, compared to the reference cohort (1937), persons born in 1962 had a 1.65-fold (95% CI = 1.19–2.27) increased risk of dying of sCJD, while those born in 1922 had a 0.60-fold (95% CI = 0.51–0.69) reduced risk. Alternatively, when the cohort effect was constrained to be 0 on average with 0 slope, mortality rates increased from periods 1992 to 2007 and remained stable thereafter (Figure S3).

**Table 1:** Age-period-cohort analysis: assessment of the goodness-of-fit of the models

Terms	DF	Deviance	Deviance/DF	AIC	Comparison of models	*P*
A + P	80	103.3	1.29	548	A + P vs. A + P + C	0.002
A + C	78	106.7	1.37	555	A + C vs. A + P + C	< 0.001
A + P + C	76	86.5	1.14	539	.	.
A + P + C + S + A×S	70	70.0	1.00	534	A + P + C + S + A×S vs. A + P + C	0.012

DF, degrees of freedom; AIC, Akaike’s information criterion; A, Age; P, Period; C, Cohort; S, sex; A×S, interaction between age and sex

Results of sensitivity analyses based on alternative parametrizations of the APC model and restricted to the M1 sCJD subgroup are presented in supplementary (Supplementary Results).

## Discussion

The present study analyzed a large dataset collected over 25-years of active national surveillance in France and aimed at exploring trends in sCJD mortality rates over time and the effect of sex, age at death, period of death and cohort of birth on sCJD mortality. Our analyses are in favour of sex, age, period, and cohort effects on sCJD mortality.

As previously reported, mortality rates reached a peak between 75 and 79 years and decreased thereafter in both sexes [19–22]. This observation is in contrast with other dementias, particularly Alzheimer disease (AD), for which incidence increases progressively with age [23]. The reasons for decreasing sCJD mortality rates in the eldest are unknown. It has been hypothesized that it can be related to under-ascertainment of sCJD in the elderly due to higher frequency of atypical phenotypes [24] that may preclude an accurate diagnosis [25]. However, under-ascertainment would need to be quite large to explain this pattern, whereas neuropathological studies in brain banks of patients with a diagnosis of dementia identified a proportion of undiagnosed CJD cases comprised between 0 and 1.3% [26–29]. Another hypothesis comes from amyotrophic lateral sclerosis (ALS), another proteinopathy, with a similar pattern of declining mortality rates after 80 years. Gompertzian analyses suggested that decline in mortality rates from ALS in the elderly may be explained by the existence of a susceptible population to which ALS deaths are limited and whose frequency has decreased over time [30, 31]. This model predicts that mortality rates decrease over time in younger age groups while they increase at older ages; however, in our data, mortality increased with time independently of age.

We report for the first time an interaction between sex and age, with a shift in M/F mortality ratios before and after the peak in mortality rates at 75–79 years. Mortality rates were higher in women than in men before this age, while the opposite was observed at older ages. This interaction was not statistically significant in analyses restricted to the M1 strain. However, the relative small size of M1 sCJD group potentially resulted in a lack of statistical power. A gender effect has been reported in iatrogenic CJD after treatment with human cadaver-sourced growth hormone (hGH-iCJD) and in vCJD cases [32–34], raising the question of the influence of sex on prion propagation and incubation, in particular after peripheral exposure as suggested by experimental transmission models. Indeed, a sex-difference in the incubation period was reported in animal models, suggesting an influence of sexual hormones [33, 35, 36]. The age-dependent sex effect seen in our sCJD population could be explained by similar processes. Since a sex effect has already been observed in peripherally transmitted forms (vCJD, hGH-iCJD, experimental infection models), our results are compatible with an exogenous origin for at least some sCJD cases with a longer incubation period in men. Another explanation could be a joint effect of sex and age on stochastic PrP conversion through unknown mechanisms that could be experimentally investigated [37].

Our APC analyses showed that the model that best fitted the data was the full model including age, period, and cohort effects; this pattern was confirmed in analyses restricted to the M1 strain. The strongest period effect was seen at the beginning of the study and mortality stabilized after this initial period; this is likely due to the implementation of the surveillance network and improvement of case identification over the first years of surveillance [13, 14, 38]. The identification of a cohort effect is in favour of an environmental factor that could influence sCJD susceptibility across birth cohorts. Variations in the frequency and intensity of exposure to potential environmental risk factors across birth cohorts may account for the observed differences in mortality in our study. This cohort effect was observed graphically and confirmed statistically through APC analyses. It appeared to be rather continuous across successive birth cohorts. In addition, the lack of significant interaction between sex and the cohort effect suggest that cohort effects are similar for men and women.

Only one previous study used an APC analysis based on 716 patients identified in the United Kingdom (1970–1997) [16]. This study considered AP and AC models, but not a full APC model, and showed that both models had a good fit; however, the AIC value was lower for the AC (23.93) than for the AP (29.05) model, thus suggesting a better fit for the AC model. We used a more sophisticated APC modelling approach, including a full APC parametrization, based on considerably larger sample size.

Besides risk factors explored in case-control studies, the possibility of zoonotic risk factors remains a possibility that could account for an exogenous origin in some sCJD cases. Research on atypical forms of BSE (L-BSE, H-BSE) has revealed molecular similarities between the L-BSE strain and molecular subtypes of human sCJD, in particular the MV2 subtype [39]. Furthermore, L-BSE has been experimentally transmitted to non-human primates as efficiently as classical BSE responsible for vCJD in humans, and could be even more virulent [40–42]. The zoonotic risk associated with natural sheep scrapie has also been recently updated with the demonstration of an intracerebral transmission of scrapie to mice expressing the human prion protein during serial passages, as well as transmission of scrapie to primates. These observations highlight the possibility of a causal link between exposure to sheep scrapie and sCJD in some cases [43, 44]. A large increase in animal product consumption and the generalization of mechanically separated meat in developed countries over the last century may have contribute to increase the zoonotic prion pressure [45]. It would be of interest to observe the effect of safety measures implemented since the “mad cow crisis” to avoid population prion exposure on sCJD mortality in the next decades.

Other environmental factors may be involved in sCJD occurrence by influencing spontaneous protein misfolding or abnormal prion protein propagation. For instance, important changes in agricultural and medical practices have occurred during the twentieth century, including widespread use of pesticides and antibiotics; both categories of products have been proposed as risk factors for other brain proteinopathies, including AD [46] and Parkinson’s disease (PD) [47–52].

The main strength of our study is the long period of CJD data collection based on an active surveillance system that provided a large sample size to study a rare disease and allowed to implement an APC model. Our study has also limitations. Exclusion of patients under 45 years of age due to the low number of cases could limit the observation of atypical sCJD cases due to environmental exposures. For instance, vCJD due to a contamination by the agent of classical BSE occurred mostly in young people (median age of onset of 27 years in the UK and 35 in France) [53, 54]. In addition, we were not able to perform separate analyses for strains other than M1 due to the small number of cases.

Based on 25 years of active surveillance in France, we show evidence for sex, age, period, and cohort effects on sCJD mortality. The identification of cohort effects suggests that environmental exposures may play a role in sCJD etiology. Our findings warrant replication in other countries, as well as further studies focusing on the identification of environmental factors associated with sCJD mortality.

## Electronic supplementary material

Below is the link to the electronic supplementary material.

Supplementary Material 1

## References

[1] Aguzzi A, Sigurdson C, Heikenwaelder M (2008). Molecular mechanisms of prion pathogenesis. Annu Rev Pathol.

[2] Prusiner SB, Prions (1998). Proc Natl Acad Sci U S A.

[3] Bishop MT, Will RG, Manson JC (2010). Defining sporadic Creutzfeldt-Jakob disease strains and their transmission properties. Proc Natl Acad Sci U S A.

[4] Laske C, Gefeller O, Pfahlberg A, Zerr I, Schröter A, Poser S (1999). The effect of stress on the onset and progression of Creutzfeldt-Jakob disease: results of a german pilot case-control study. Eur J Epidemiol.

[5] Collins S, Law MG, Fletcher A, Boyd A, Kaldor J, Masters CL (1999). Surgical treatment and risk of sporadic Creutzfeldt-Jakob disease: a case-control study. Lancet.

[6] Ruegger J, Stoeck K, Amsler L (2009). A case-control study of sporadic Creutzfeldt-Jakob disease in Switzerland: analysis of potential risk factors with regard to an increased CJD incidence in the years 2001–2004. BMC Public Health.

[7] van Duijn CM, Delasnerie-Laupretre N, Masullo C (1998). Case-control study of risk factors of Creutzfeldt-Jakob disease in Europe during 1993-95. European Union (EU) collaborative Study Group of Creutzfeldt-Jakob disease (CJD). Lancet.

[8] Ward HJ, Everington D, Croes EA (2002). Sporadic Creutzfeldt-Jakob disease and surgery: a case-control study using community controls. Neurology.

[9] Mahillo-Fernandez I, de Pedro-Cuesta J, Bleda MJ (2008). Surgery and risk of sporadic Creutzfeldt-Jakob disease in Denmark and Sweden: registry-based case-control studies. Neuroepidemiology.

[10] de Pedro-Cuesta J, Mahillo-Fernández I, Rábano A (2011). Nosocomial transmission of sporadic Creutzfeldt-Jakob disease: results from a risk-based assessment of surgical interventions. J Neurol Neurosurg Psychiatry.

[11] Molesworth AM, Mackenzie J, Everington D, Knight RS, Will RG. Sporadic Creutzfeldt-Jakob disease and risk of blood transfusion in the United Kingdom. Transfusion. 2011;51(8):1872-3; author reply 3–4. 10.1111/j.1537-2995.2011.03198.x 10.1111/j.1537-2995.2011.03198.x 21831186

[12] de Pedro Cuesta J, Ruiz Tovar M, Ward H (2012). Sensitivity to biases of case-control studies on medical procedures, particularly surgery and blood transfusion, and risk of Creutzfeldt-Jakob disease. Neuroepidemiology.

[13] Uttley L, Carroll C, Wong R, Hilton DA, Stevenson M (2020). Creutzfeldt-Jakob disease: a systematic review of global incidence, prevalence, infectivity, and incubation. Lancet Infect Dis.

[14] Klug GM, Wand H, Simpson M (2013). Intensity of human prion disease surveillance predicts observed disease incidence. J Neurol Neurosurg Psychiatry.

[15] Carstensen B (2007). Age–period–cohort models for the Lexis diagram. Stat Med.

[16] Cohen CH (2000). Does improvement in case ascertainment explain the increase in sporadic Creutzfeldt-Jakob disease since 1970 in the United Kingdom?. Am J Epidemiol.

[17] Rutherford MJ, Lambert PC, Thompson JR (2010). Age–period–cohort modeling. Stata J.

[18] Clayton D, Schifflers E (1987). Models for temporal variation in cancer rates. I: age–period and age–cohort models. Stat Med.

[19] Sun Y, Liu CC, Fan LY (2020). Incidence of and Mortality due to Human Prion Diseases in Taiwan: a prospective 20-Year Nationwide Surveillance Study from 1998 to 2017. Clin Epidemiol.

[20] Coulthart MB, Jansen GH, Connolly T et al. Creutzfeldt-Jakob disease mortality in Canada, 1998 to 2013. Canada communicable disease report = Releve des maladies transmissibles au Canada. 2015;41(8):182–91. 10.14745/ccdr.v41i08a01 10.14745/ccdr.v41i08a01 PMC5864311 29769950

[21] Nozaki I, Hamaguchi T, Sanjo N (2010). Prospective 10-year surveillance of human prion diseases in Japan. Brain.

[22] Cousens SN, Zeidler M, Esmonde TF et al. Sporadic Creutzfeldt-Jakob disease in the United Kingdom: analysis of epidemiological surveillance data for 1970-96. BMJ (Clinical research ed.). 1997;315(7105):389–95. 10.1136/bmj.315.7105.389 10.1136/bmj.315.7105.389 PMC2127296 9277601

[23] Niu H, Álvarez-Álvarez I, Guillén-Grima F, Aguinaga-Ontoso I (2017). Prevalence and incidence of Alzheimer’s disease in Europe: a meta-analysis. Neurologia.

[24] Heinemann U, Krasnianski A, Meissner B (2007). Creutzfeldt-Jakob disease in Germany: a prospective 12-year surveillance. Brain.

[25] Plaitakis A, Viskadouraki AK, Tzagournissakis M (2001). Increased incidence of sporadic Creutzfeldt-Jakob disease on the island of Crete associated with a high rate of PRNP 129-methionine homozygosity in the local population. Ann Neurol.

[26] Jellinger K, Danielczyk W, Fischer P, Gabriel E (1990). Clinicopathological analysis of dementia disorders in the elderly. J Neurol Sci.

[27] Mendez MF, Mastri AR, Sung JH, Frey WH (1992). 2nd. Clinically diagnosed Alzheimer disease: neuropathologic findings in 650 cases. Alzheimer Dis Assoc Disord.

[28] Barker WW, Luis CA, Kashuba A (2002). Relative frequencies of Alzheimer disease, Lewy body, vascular and frontotemporal dementia, and hippocampal sclerosis in the state of Florida Brain Bank. Alzheimer Dis Assoc Disord.

[29] Beach TG, Adler CH, Sue LI (2015). Arizona study of aging and neurodegenerative Disorders and Brain and Body Donation Program. Neuropathology: official journal of the Japanese Society of Neuropathology.

[30] Riggs JE, the U.S. Longitudinal Gompertzian analysis of amyotrophic lateral sclerosis mortality in, 1977–1986: evidence for an inherently susceptible population subset. Mech Ageing Dev. 1990;55(3):207 – 20. 10.1016/0047-6374(90)90149-a 10.1016/0047-6374(90)90149-a 2232913

[31] Neilson S, Robinson I, Alperovitch A (1994). Rising amyotrophic lateral sclerosis mortality in France 1968–1990: increased life expectancy and inter-disease competition as an explanation. J Neurol.

[32] Peckeu L, Brandel JP, Welaratne A, Costagliola D, Haïk S (2018). Susceptibility to Creutzfeldt-Jakob disease after human growth hormone treatment in France. Neurology.

[33] Loeuillet C, Boelle PY, Lemaire-Vieille C (2010). Sex effect in mouse and human prion disease. J Infect Dis.

[34] Nishimura Y, Harada K, Koyama T, Hagiya H, Otsuka F (2020). A nationwide trend analysis in the incidence and mortality of Creutzfeldt-Jakob disease in Japan between 2005 and 2014. Sci Rep.

[35] Kimberlin RH, Walker C (1977). Characteristics of a short incubation model of scrapie in the golden hamster. J Gen Virol.

[36] Outram GW (1976). The pathogenesis of scrapie in mice. Front Biol.

[37] Watts JC, Giles K, Stöhr J (2012). Spontaneous generation of rapidly transmissible prions in transgenic mice expressing wild-type bank vole prion protein. Proc Natl Acad Sci U S A.

[38] Watson N, Brandel JP, Green A et al. The importance of ongoing international surveillance for Creutzfeldt-Jakob disease.Nature reviews. Neurology. 2021:1–18. 10.1038/s41582-021-00488-7 10.1038/s41582-021-00488-7 PMC8109225 33972773

[39] Casalone C, Zanusso G, Acutis P (2004). Identification of a second bovine amyloidotic spongiform encephalopathy: molecular similarities with sporadic Creutzfeldt-Jakob disease. Proc Natl Acad Sci U S A.

[40] Comoy EE, Casalone C, Lescoutra-Etchegaray N (2008). Atypical BSE (BASE) transmitted from asymptomatic aging cattle to a primate. PLoS ONE.

[41] Ono F, Tase N, Kurosawa A (2011). Atypical L-type bovine spongiform encephalopathy (L-BSE) transmission to cynomolgus macaques, a non-human primate. Jpn J Infect Dis.

[42] Mestre-Francés N, Nicot S, Rouland S (2012). Oral transmission of L-type bovine spongiform encephalopathy in primate model. Emerg Infect Dis.

[43] Cassard H, Torres JM, Lacroux C (2014). Evidence for zoonotic potential of ovine scrapie prions. Nat Commun.

[44] Comoy EE, Mikol J, Luccantoni-Freire S (2015). Transmission of scrapie prions to primate after an extended silent incubation period. Sci Rep.

[45] Chalus T, Peutz I. BSE: the European regulatory context. Euro surveillance : bulletin Europeen sur les maladies transmissibles = European communicable disease bulletin. 2000;5(10):107–12. 10.2807/esm.05.10.00002-en 10.2807/esm.05.10.00002-en 12631966

[46] Angelucci F, Cechova K, Amlerova J, Hort J (2019). Antibiotics, gut microbiota, and Alzheimer’s disease. J Neuroinflammation.

[47] Romano S, Savva GM, Bedarf JR, Charles IG, Hildebrand F, Narbad A (2021). Meta-analysis of the Parkinson’s disease gut microbiome suggests alterations linked to intestinal inflammation. NPJ Parkinsons Dis.

[48] Ternak G, Kuti D, Kovacs KJ (2020). Dysbiosis in Parkinson’s disease might be triggered by certain antibiotics. Med Hypotheses.

[49] Shrestha S, Parks CG, Umbach DM (2020). Pesticide use and incident Parkinson’s disease in a cohort of farmers and their spouses. Environ Res.

[50] Ahmed H, Abushouk AI, Gabr M, Negida A, Abdel-Daim MM (2017). Parkinson’s disease and pesticides: a meta-analysis of disease connection and genetic alterations. Biomed Pharmacother.

[51] Elbaz A, Carcaillon L, Kab S, Moisan F (2016). Epidemiology of Parkinson’s disease. Rev Neurol (Paris).

[52] Ball N, Teo WP, Chandra S, Chapman J (2019). Parkinson’s Disease and the Environment. Front Neurol.

[53] Unit NCRS. Creutzfeldt-Jakob Disease Surveillance in the UK. 28th Annual Report 2019. http://www.cjd.ed.ac.uk/sites/default/files/Report28.pdf.

[54] Brandel JP, Heath CA, Head MW (2009). Variant Creutzfeldt-Jakob disease in France and the United Kingdom: evidence for the same agent strain. Ann Neurol.

