# Supplementary material for: The role of environmental factors on sporadic Creutzfeldt-Jakob disease mortality: evidence from an age-period-cohort analysis
Source: Eur J Epidemiol. 2023 May 16;38(7):757–64. doi: 10.1007/s10654-023-01004-5 (PMC10276107; doi:10.1007/s10654-023-01004-5)
Supplement: Supplementary file 1 — Supplementary Material 1 [file 10654_2023_1004_MOESM1_ESM.pdf]

Supplemental material

The role of environmental factors on sporadic Creutzfeldt-Jakob disease mortality: Evidence from an age-period-cohort analysis

Author(s) :

Angéline Denouel, PhD[1], Jean-Philippe Brandel, MD[1,2], Danielle Seilhean, MD, PhD[1], Jean-Louis Laplanche, PhD[3,4], Alexis Elbaz, MD, PhD[5†], Stéphane Haik, MD, PhD[1,2†]

[†]These authors share senior authorship.

Corresponding Author:

Angéline Denouel, angeline.denouel@icm-institute.org

Affiliation Information for All Authors:

1. CNRS UMR 7225, INSERM U1127, Paris Brain Institute, Sorbonne Universités, Paris, France ; 2. AP-HP, Centre National de Référence des Maladies de Creutzfeldt-Jakob, Groupe Hospitalier Pitié-Salpêtrière, Paris, France ; 3. Département de Biochimie et Biologie Moléculaire, Hôpitaux Lariboisière-Fernand Widal, Paris, France ; 4. INSERM, UMR 1144, "Optimisation Thérapeutique en Neuropsychopharmacologie", Paris, France ; 5. Université Paris-Saclay, UVSQ, Univ. Paris-Sud, Gustave Roussy, Inserm, U1018, Team « Exposome, Heredity, Cancer, and Health », CESP, 94807, Villejuif, France.

Supplementary Methods

French National CJD Surveillance Network

Following the emergence of the first cases of iatrogenic CJD after growth hormone treatment and the risk of interspecies transmission of BSE, a surveillance network was established in 1991 in France and is currently coordinated by the French agency for health surveillance (*Santé Publique France*). As part of this network, a secured and anonymous database centralizes demographical, clinical, genetic, neuropathological, and biochemical data collected for each suspected case of TSE.

The majority of suspected cases are notified to the network by biochemistry laboratories performing 14-3-3 protein detection in cerebrospinal fluid (CSF). Other notifications are made directly by neurologists, other physicians, or, more rarely, neuropathological laboratories. All suspected cases are followed until a final diagnosis is obtained (TSE or other diagnosis). TSE cases are classified as possible, probable or certain by etiology, according to the diagnostic criteria from the European "EuroCJD" network. For sCJD, the classification of cases has followed the evolution of the criteria: probable case defined by suggestive clinical signs and a characteristic EEG before 1998; characteristic EEG or detection of 14-3 -3 protein in the CSF from 1998 to 2010; and characteristic EEG, detection of 14-3-3 protein or high signals in caudate/putamen on MRI brain scan since 2010 [1]. A diagnosis of definite TSE is made exclusively by a pathological examination of brain (autopsy or biopsy).

Molecular subtypes and strains

In patients for whom we obtained a blood sample and extracted DNA, we obtained written informed consent to genotype the codon 129 polymorphism (M129V; M, methionine; V, valine) of the prion protein gene [2]. In the general population, 50% of persons are homozygous (MM or VV) and 50% heterozygous whereas 80% of sCJD cases carry the MM genotype [3].

Written consent from relatives was requested to perform autopsies after patients' death. We classified the protease-resistant protein (PrP^{Sc}) type observed in frozen samples from the brain [4]. After proteinase K digestion, PrP^{Sc} shows 3 bands in Western-blots corresponding to di-, mono-, and non-glycosylated PrP forms (36, 30, and 19-21kDa migrations respectively). In sCJD, the size of the non-glycosylated band enables to distinguish type 1 (molecular mass of 21kDa) from type 2 (molecular mass of 19kDa).

The "Cleveland classification"[4], combining codon 129 genotypes and PrP^{Sc} typing, defined 6 molecular subtypes (MM1/MV1, MV2, VV2, VV1, MM2-cortical, MM2-thalamic) corresponding to different sCJD clinico-pathological phenotypes and strains with specific characteristics in terms of incubation time, distribution of brain lesions, or location of abnormal PrP in the brain [5]. MM1 and MV1 subtypes demonstrate similar properties and correspond to the M1 strain, which is the most frequent. MV2 and VV2 correspond to the V2 strain and VV1 to the V1 strain. From MM2-cortical and MM2-thalamic cases, two distinct strains were isolated [6].

Because the autopsy rate of sCJD cases was limited and in order to increase the size of the M1 sCJD group, we aimed at classifying non-autopsied patients on the basis of clinical data. First, we used data from patients with a codon 129 genotype and a neuropathological examination of the brain including PrP^{Sc} typing by Western-blot to determine the strain. We then used the clinical information available for these patients at presentation and death (abnormal 14-3-3 level, dementia, myoclonus, cerebellar syndrome, visual symptoms, pyramidal and extra-pyramidal symptoms, mutism) to build a prediction model that is able to distinguish the M1 strain (N=363) from all others (N=266); the prediction of strains other than M1 was not possible due to insufficient sample sizes. We first used multivariable logistic regression to select the subset of clinical characteristics significantly and independently associated with M1 status at $P\leq 0.05$ (age at disease onset, disease duration, dementia, pyramidal symptoms at disease onset, myoclonus at death). We then applied nonparametric applied k^{th}-nearest-neighbor discriminant analysis based on the variables previously selected, and used this model to predict M1 status in patients for whom the genetic test and/or neuropathological examination of brain were not available. This algorithm enabled to distinguish the M1 strain from other strains with 67% sensitivity and 100% specificity.

Supplementary Results

Alternative parametrizations of the age-period-cohort model

In sensitivity analyses using the three time variables as factors, we constrained the regression coefficients of the two last periods of deaths (2002-2006, 2007-2012) to be equal and reached similar conclusions (Table S5; Figure S4). The full APC model had a better fit compared to the A ($P<0.001$), AD ($P<0.001$), AP ($P=0.040$), and AC ($P<0.001$) models. In addition, including an interaction between age and sex in the APC model provided a better fit to the data compared to the APC model without the interaction ($P=0.027$).

Analyses restricted to sCJD cases with the M1 strain

Analyses restricted to sCJD cases with the M1 strain are based on 972 cases (438 men, 534 women; 39% of all sCJD patients in this age group) aged 45-89 years old. M1 status was predicted by the algorithm for 609 (63%) of them. The overall mortality rate was 1.91 per 1,000,000 person-years (95% CI=1.78-2.04; Table S6). The age-standardized mortality rate was 1.86 per 1,000,000 person-years (95% CI=1.69-2.02) in women and 1.99 per 1,000,000 person-years (95% CI=1.79-2.20) in men. Unlike analyses based on all sCJD cases, there was no significant interaction between age and sex (P=0.550), although age-specific rates tended to be higher in men than women after 80 years (Figure S5). APC analyses restricted to the M1 strain showed that the full APC model without the interaction between age and sex had the smallest AIC value and provided the best fit to the data (Table S7).

Table S1. Age-specific mortality rates of sCJD per 1,000,000 person-years, overall and by sex (France, 1992-2016).

	Men		Women		Overall	
**Age at death**	**N**	**Mortality rate (95% CI)**	**N**	**Mortality rate (95% CI)**	**N**	**Mortality rate (95% CI)**
**45-49**	12	0.229 (0.099-0.358)	26	0.486 (0.299-0.673)	38	0.359 (0.245-0.473)
**50-54**	40	0.830 (0.573-1.087)	67	1.356 (1.031-1.681)	107	1.096 (0.889-1.304)
**55-59**	102	2.344 (1.889-2.799)	115	2.538 (2.075-3.002)	217	2.443 (2.118-2.768)
**60-64**	161	4.141 (3.501-4.780)	192	4.592 (3.943-5.242)	353	4.375 (3.918-4.831)
**65-69**	255	7.640 (6.702-8.578)	267	7.014 (6.173-7.856)	522	7.307 (6.680-7.934)
**70-74**	208	7.630 (6.593-8.667)	276	8.081 (7.128-9.035)	484	7.881 (7.179-8.583)
**75-79**	191	9.181 (7.879-10.483)	260	8.826 (7.753-9.899)	451	8.973 (8.145-9.801)
**80-84**	103	7.171 (5.786-8.556)	140	5.792 (4.833-6.751)	243	6.306 (5.513-7.099)
**85-89**	25	3.273 (1.990-4.556)	35	2.157 (1.442-2.872)	60	2.514 (1.878-3.150)
**Total**	1097	4.715 (4.403-5.028)	1378	4.538 (4.290-4.786)	2475	4.584 (4.392-4.775)

CI, confidence intervals computed using Poisson regression.

Table S2. Poisson regression models for mortality of sCJD.

Characteristics	Beta	SE	Relative risk (95% CI)	*P*	*P*
**Overall mortality**					
Intercept	-0.89	0.12	0.41 (0.32-0.52)	$<10^{-3}$	
Age	1.97	0.21	7.14 (4.71-10.82)	$<10^{-3}$	
Age^2	-0.18	0.11	0.84 (0.67-1.04)	0.114	
Age^3	-0.05	0.02	0.95 (0.92-0.99)	0.009	<0.001[a]
**Mortality by sex**					
Intercept	-0.56	0.15	0.57 (0.43-0.77)	$<10^{-3}$	
Male sex[c]	-0.81	0.26	0.44 (0.27-0.74)	0.002	
Age	1.54	0.27	4.64 (2.75-7.85)	$<10^{-3}$	
Age^2	0.01	0.15	1.01 (0.76-1.34)	0.945	
Age^3	-0.08	0.02	0.93 (0.89-0.97)	0.001	<0.001[a]
Male sex × Age	1.10	0.44	2.99 (1.26-7.11)	0.013	
Male sex × Age^2	-0.50	0.23	0.61 (0.39-0.96)	0.033	
Male sex × Age^3	0.08	0.04	1.08 (1.00-1.16)	0.040	0.001[b]

SE, standard error.
Relative risks are the exponentials of the regression coefficients beta.
Figures 1 and 3 provide a graphical representation of these models.
Age is centered at 45 years and divided by 10.
[a] Global test for age.
[a] Global test for the interaction between sex and age.
[c] The relative risk for male sex compares mortality in men to mortality in women at the age of 45 years.

Table S3. Standardized mortality rates (SMR per 1,000,000 person-years) of sCJD by year of death, overall and by sex (France, 1992-2016).

Year of	Men			Women			Overall		
death	N	Crude rate	SMR[a] (95% CI)	N	Crude rate	SMR[a] (95% CI)	N	Crude rate	SMR[b] (95% CI)
**1992**	16	0.574	0.655 (0.654-0.656)	22	0.749	0.774 (0.773-0.775)	38	0.664	0.727 (0.727-0.728)
**1993**	13	0.465	0.551 (0.550-0.552)	22	0.746	0.820 (0.819-0.821)	35	0.609	0.699 (0.698-0.700)
**1994**	20	0.713	0.903 (0.902-0.904)	25	0.844	0.803 (0.802-0.804)	45	0.781	0.840 (0.839-0.841)
**1995**	23	0.818	0.920 (0.919-0.921)	36	1.211	1.195 (1.194-1.196)	59	1.020	1.080 (1.079-1.080)
**1996**	36	1.277	1.535 (1.534-1.537)	32	1.073	1.116 (1.114-1.117)	68	1.172	1.282 (1.281-1.283)
**1997**	35	1.238	1.503 (1.502-1.505)	45	1.503	1.487 (1.486-1.488)	80	1.374	1.468 (1.467-1.469)
**1998**	28	0.987	1.093 (1.091-1.094)	53	1.765	1.693 (1.692-1.695)	81	1.387	1.442 (1.441-1.443)
**1999**	43	1.510	1.803 (1.802-1.805)	49	1.623	1.580 (1.579-1.582)	92	1.568	1.666 (1.665-1.667)
**2000**	43	1.501	1.758 (1.756-1.760)	45	1.480	1.488 (1.487-1.490)	88	1.490	1.572 (1.571-1.573)
**2001**	53	1.837	2.174 (2.172-2.176)	56	1.828	1.700 (1.698-1.701)	109	1.833	1.879 (1.878-1.880)
**2002**	51	1.757	2.095 (2.093-2.097)	56	1.815	1.768 (1.767-1.770)	107	1.787	1.870 (1.869-1.871)
**2003**	45	1.540	1.731 (1.730-1.733)	63	2.027	1.832 (1.830-1.833)	108	1.791	1.791 (1.790-1.792)
**2004**	47	1.598	1.773 (1.772-1.775)	51	1.628	1.520 (1.519-1.521)	98	1.614	1.616 (1.615-1.617)
**2005**	37	1.249	1.379 (1.377-1.380)	45	1.426	1.336 (1.335-1.337)	82	1.340	1.339 (1.338-1.340)
**2006**	44	1.476	1.599 (1.597-1.600)	80	2.517	2.261 (2.259-2.262)	124	2.013	1.983 (1.982-1.985)
**2007**	55	1.833	2.029 (2.027-2.031)	83	2.597	2.377 (2.375-2.378)	138	2.227	2.203 (2.202-2.204)
**2008**	49	1.624	1.737 (1.735-1.738)	56	1.743	1.629 (1.628-1.631)	105	1.685	1.674 (1.673-1.675)
**2009**	51	1.682	1.760 (1.758-1.761)	63	1.951	1.742 (1.741-1.743)	114	1.821	1.746 (1.745-1.747)
**2010**	64	2.100	2.229 (2.227-2.230)	87	2.682	2.398 (2.397-2.400)	151	2.400	2.324 (2.323-2.325)
**2011**	56	1.829	1.892 (1.890-1.894)	59	1.810	1.609 (1.608-1.611)	115	1.819	1.704 (1.703-1.705)
**2012**	61	1.982	2.019 (2.018-2.021)	70	2.137	1.913 (1.912-1.915)	131	2.062	1.946 (1.945-1.947)
**2013**	60	1.939	1.855 (1.854-1.857)	64	1.944	1.734 (1.733-1.735)	124	1.942	1.801 (1.800-1.802)
**2014**	63	2.027	1.982 (1.981-1.984)	87	2.630	2.295 (2.294-2.297)	150	2.338	2.136 (2.135-2.137)
**2015**	62	1.988	1.877 (1.875-1.878)	69	2.078	1.714 (1.712-1.715)	131	2.035	1.798 (1.797-1.799)
**2016**	57	1.824	1.746 (1.744-1.747)	80	2.402	2.044 (2.043-2.046)	137	2.122	1.901 (1.900-1.902)

N, number of sCJD cases; CI, confidence interval.

[a] Age-standardized mortality rates using the French population as the reference.

[b] Age- and sex-standardized mortality rates using the French population (1992-2016) as the reference.

Table S4. Mortality rates (per 1,000,000 person-years) of sCJD by sex, age group, and period of death (France, 1992-2016).

	Period of death									
Age	1992-1996		1997-2001		2002-2006		2007-2011		2012-2016	
group	N	MR (95% CI)	N	MR (95% CI)	N	MR (95% CI)	N	MR (95% CI)	N	MR (95% CI)
**Men**										
**45-49**	2	0.199 (0.024-0.717)	2	0.191 (0.023-0.688)	3	0.288 (0.059-0.843)	4	0.373 (0.102-0.955)	1	0.093 (0.002-0.516)
**50-54**	3	0.413 (0.085-1.207)	6	0.612 (0.225-1.332)	9	0.873 (0.399-1.658)	7	0.684 (0.275-1.409)	15	1.418 (0.794-2.338)
**55-59**	20	2.888 (1.764-4.461)	15	2.140 (1.198-3.529)	19	1.988 (1.197-3.104)	26	2.593 (1.694-3.799)	22	2.203 (1.381-3.335)
**60-64**	19	2.749 (1.655-4.292)	21	3.220 (1.993-4.922)	35	5.216 (3.633-7.254)	36	3.938 (2.758-5.452)	50	5.211 (3.867-6.870)
**65-69**	27	4.355 (2.870-6.337)	52	8.264 (6.172-10.837)	37	6.109 (4.301-8.420)	62	9.912 (7.599-12.706)	77	8.983 (7.089-11.227)
**70-74**	19	3.741 (2.252-5.842)	46	8.52 (6.238-11.365)	56	9.989 (7.545-12.971)	41	7.499 (5.381-10.173)	46	8.057 (5.898-10.746)
**75-79**	7	2.625 (1.056-5.409)	35	8.463 (5.894-11.769)	37	8.243 (5.804-11.362)	62	13.018 (9.981-16.689)	50	10.527 (7.813-13.878)
**80-84**	8	3.504 (1.513-6.904)	18	9.459 (5.606-14.949)	20	6.573 (4.015-10.152)	26	7.638 (4.989-11.191)	31	8.311 (5.647-11.796)
**85-89**	0	-	4	3.117 (0.849-7.981)	7	6.253 (2.514-12.884)	6	3.190 (1.171-6.943)	8	3.650 (1.576-7.193)
**Overall**	105	2.162 (1.768-2.617)	199	3.766 (3.261-4.327)	223	3.893 (3.398-4.438)	270	4.362 (3.857-4.915)	300	4.551 (4.051-5.096)
**Women**										
**45-49**	3	0.302 (0.062-0.883)	4	0.375 (0.102-0.961)	4	0.371 (0.101-0.949)	9	0.813 (0.372-1.544)	6	0.543 (0.199-1.182)
**50-54**	7	0.972 (0.391-2.002)	12	1.220 (0.631-2.132)	16	1.501 (0.858-2.438)	16	1.492 (0.853-2.423)	16	1.456 (0.832-2.365)
**55-59**	15	2.092 (1.171-3.450)	17	2.387 (1.390-3.821)	24	2.442 (1.564-3.633)	27	2.554 (1.683-3.716)	32	3.016 (2.063-4.258)
**60-64**	30	3.938 (2.657-5.622)	35	4.975 (3.465-6.919)	27	3.811 (2.512-5.545)	51	5.266 (3.921-6.924)	49	4.717 (3.490-6.236)
**65-69**	28	3.770 (2.505-5.449)	55	7.457 (5.618-9.706)	61	8.811 (6.74-11.318)	49	7.101 (5.253-9.388)	74	7.839 (6.155-9.841)
**70-74**	30	4.448 (3.001-6.349)	54	7.682 (5.771-10.023)	53	7.442 (5.575-9.735)	72	10.855 (8.493-13.670)	67	10.116 (7.840-12.847)
**75-79**	12	2.973 (1.536-5.193)	48	7.838 (5.779-10.392)	65	9.96 (7.687-12.695)	73	11.067 (8.675-13.915)	62	10.041 (7.698-12.872)
**80-84**	8	1.894 (0.818-3.733)	15	4.475 (2.505-7.381)	34	6.491 (4.495-9.070)	39	6.938 (4.934-9.485)	44	7.669 (5.573-10.296)
**85-89**	2	0.723 (0.088-2.612)	5	1.712 (0.556-3.996)	5	2.068 (0.672-4.827)	9	2.315 (1.058-4.394)	14	3.306 (1.808-5.548)
**Overall**	135	2.364 (1.982-2.798)	245	3.987 (3.503-4.519)	289	4.340 (3.854-4.871)	345	4.813 (4.318-5.348)	364	4.838 (4.354-5.361)

N, number of sCJD cases; MR, mortality rate; CI, confidence interval computed using Poisson regression.

Table S5. Age-period-cohort analysis using factor variables: assessment of the goodness-of-fit of the models.

Terms	DF	Deviance	Deviance/DF	AIC	Comparison of models	P
A	81	217.1	2.68	662	..	..
A+D	80	129.7	1.62	576	A+D *vs.* A	<0.001
A+P	77	93.7	1.22	546	A+P *vs.* A+D	<0.001
A+C	69	97.1	1.41	566	A+C *vs.* A+D	<0.001
A+P+C	66	73.3	1.11	548	A+P+C *vs.* A+P	0.040
					A+P+C *vs.* A+C	<0.001
A+P+C+S+A×S	57	54.6	0.96	547	A+P+C+S+A×S *vs.* A+P+C	0.027

DF, Degree of freedom; AIC, Akaike's information criterion; A, Age; D, drift; P, Period; C, Cohort; S, sex; A×S, interaction between age and sex.

Table S6. Age-specific mortality rates of sCJD patients with the M1 strain per 1,000,000 person-years, overall and by sex (France, 1992-2016).

	Men		Women		Overall	
**Age at death**	**N**	**Mortality rate (95% CI)**	**N**	**Mortality rate (95% CI)**	**N**	**Mortality rate (95% CI)**
**45-49**	6	0.114 (0.023-0.206)	10	0.187 (0.071-0.303)	16	0.151 (0.077-0.225)
**50-54**	7	0.145 (0.038-0.253)	10	0.202 (0.077-0.328)	17	0.174 (0.091-0.257)
**55-59**	37	0.850 (0.576-1.124)	29	0.640 (0.407-0.873)	66	0.743 (0.564-0.922)
**60-64**	64	1.646 (1.243-2.049)	76	1.818 (1.409-2.226)	140	1.735 (1.448-2.022)
**65-69**	90	2.697 (2.139-3.254)	81	2.128 (1.665-2.591)	171	2.394 (2.035-2.752)
**70-74**	67	2.458 (1.869-3.046)	96	2.811 (2.249-3.373)	163	2.654 (2.247-3.062)
**75-79**	103	4.951 (3.995-5.907)	140	4.753 (3.965-5.540)	243	4.835 (4.227-5.443)
**80-84**	54	3.760 (2.757-4.762)	74	3.061 (2.364-3.759)	128	3.322 (2.746-3.897)
**85-89**	10	1.309 (0.498-2.121)	18	1.109 (0.597-1.622)	28	1.173 (0.739-1.608)
**Total**	438	1.992 (1.785-2.200)	534	1.857 (1.694-2.020)	972	1.909 (1.782-2.036)

CI, confidence intervals computed using Poisson regression.

Table S7. Age-period-cohort analysis restricted to sCJD patients with the M1 strain: assessment of the goodness-of-fit of the models.

Terms	DF	Deviance	Deviance/DF	AIC	Comparison of models	*P*
A+P	80	105.5	1.32	460	A+P *vs*. A+P+C	0.026
A+C	78	102.2	1.31	461	A+C *vs*. A+P+C	0.020
A+P+C	76	94.4	1.24	457	..	..
A+P+C+S+A×S	70	92.1	1.32	466	A+P+C+S+A×S *vs*. A+P+C	0.894

DF, degrees of freedom; AIC, Akaike's information criterion; A, Age; P, Period; C, Cohort; S, sex; A×S, interaction between age and sex.

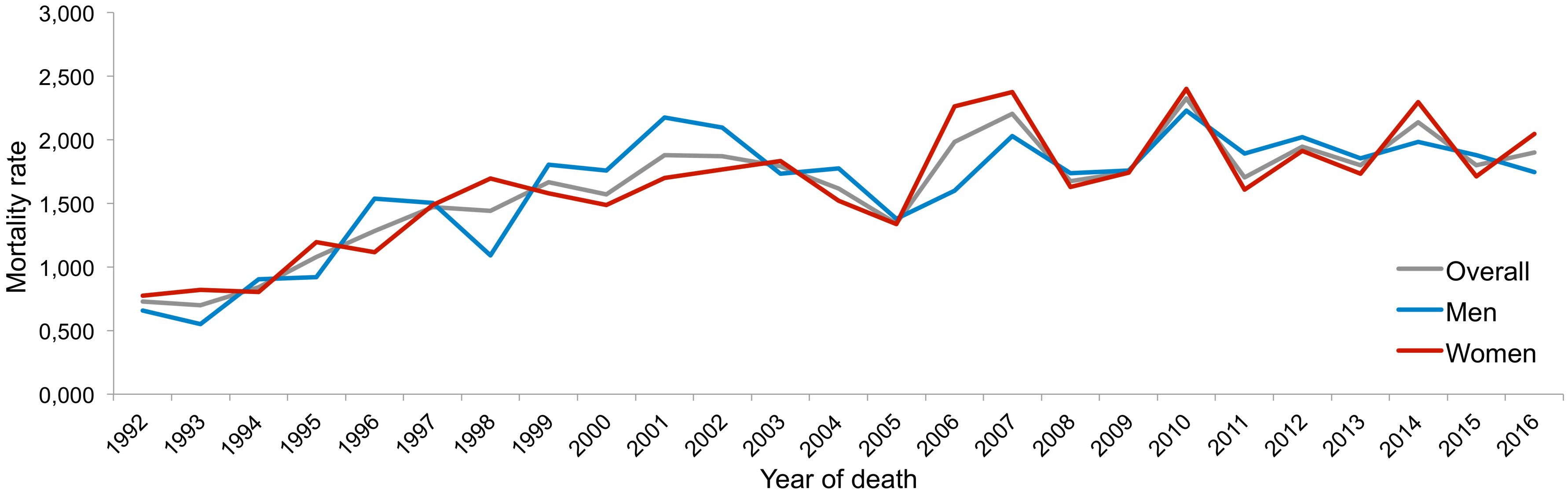

Figure S1. Standardized mortality rates (SMR per 1,000,000 person-years) of sCJD by year of death overall and by sex (France, 1992-2016).
Age- and sex-standardized mortality rate overall using the French population as reference (grey line) and age-standardized mortality rate by sex using the French population as reference (men, blue; women, red; Table S3).

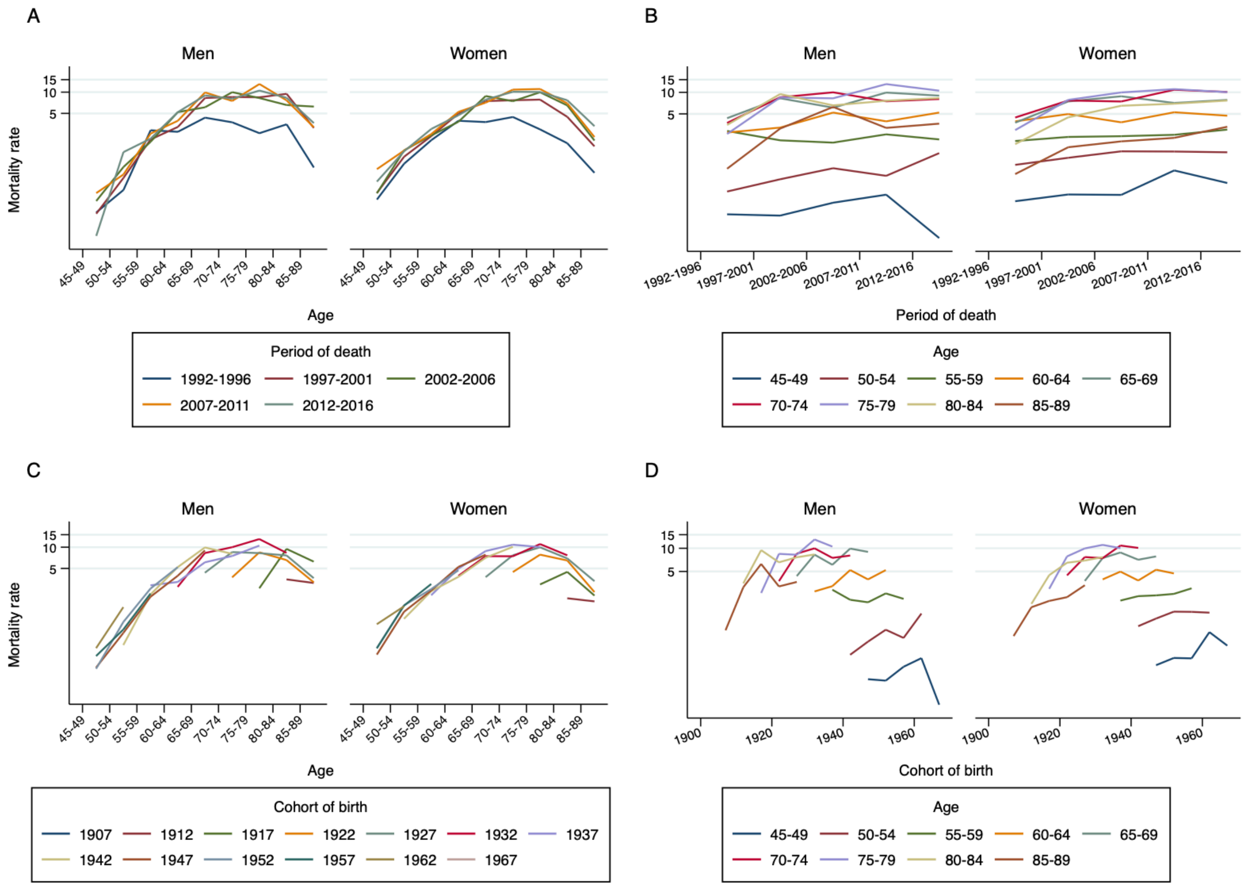

Figure S2. Mortality rates (per 1,000,000 person years) of sCJD by age group, period of death, and cohort of birth (France, 1992-2016). (A) Mortality rates of sCJD by age group, stratified by period of death. **(C)** Mortality rates of sCJD by age group, stratified by cohort of birth. **(B)** Mortality rates of sCJD by period of death, stratified by age at time of death. **(D)** Mortality rates of sCJD by cohort of birth, stratified by age. Figures use a logarithmic scale for the Y-axis.

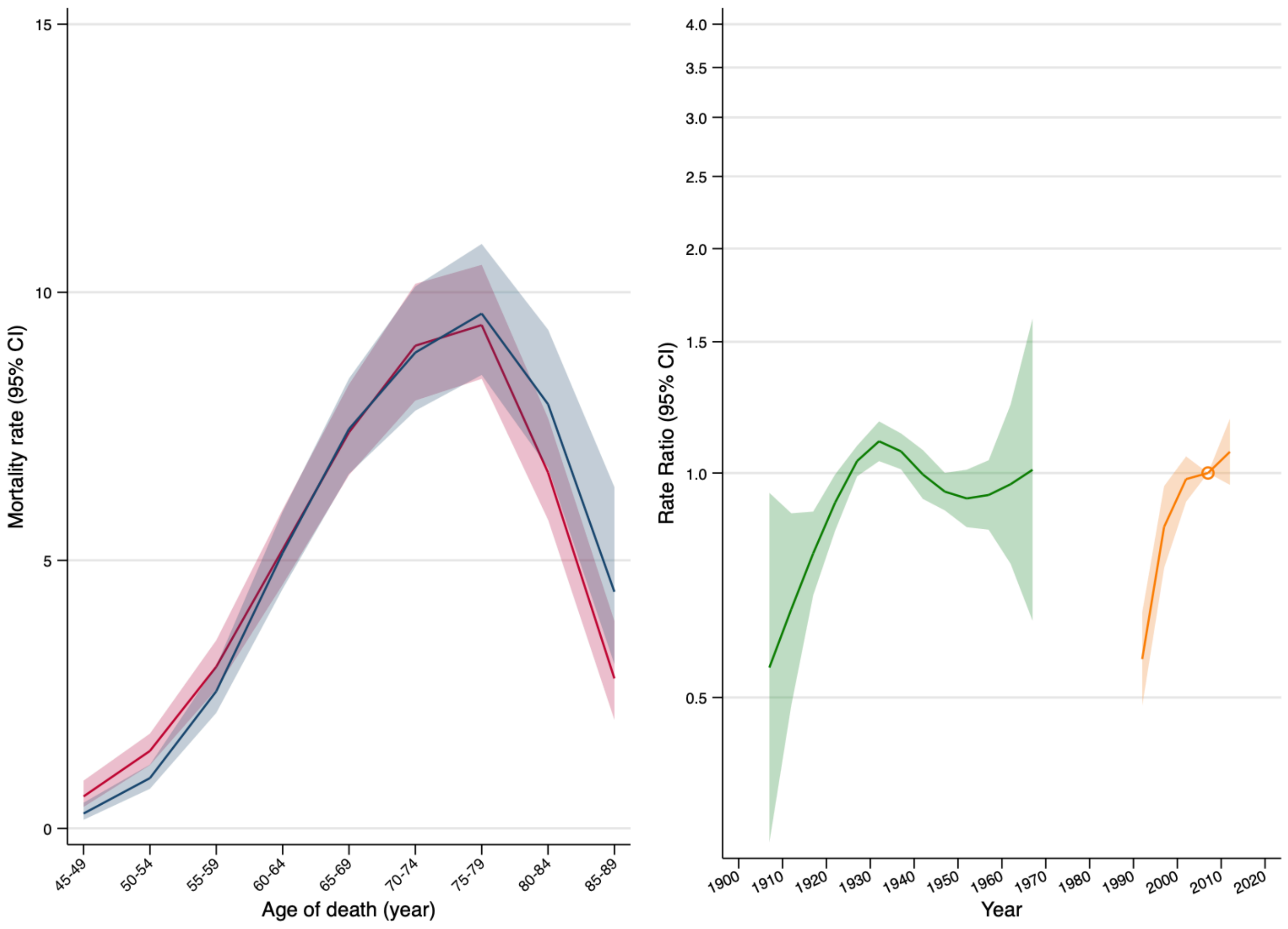

Figure S3. Age, period, and cohort effects for sCJD mortality rates: alternative parametrization.
The left part of the graph shows sCJD age-specific mortality rates (per 1,000,000 person-years; shaded area, 95% confidence intervals) in men (blue) and women (red). The right part of the graph shows relative risks (orange; shaded area, 95% confidence intervals) for periods (reference, 2007), and average cohort effects constrained to be zero on average (green).

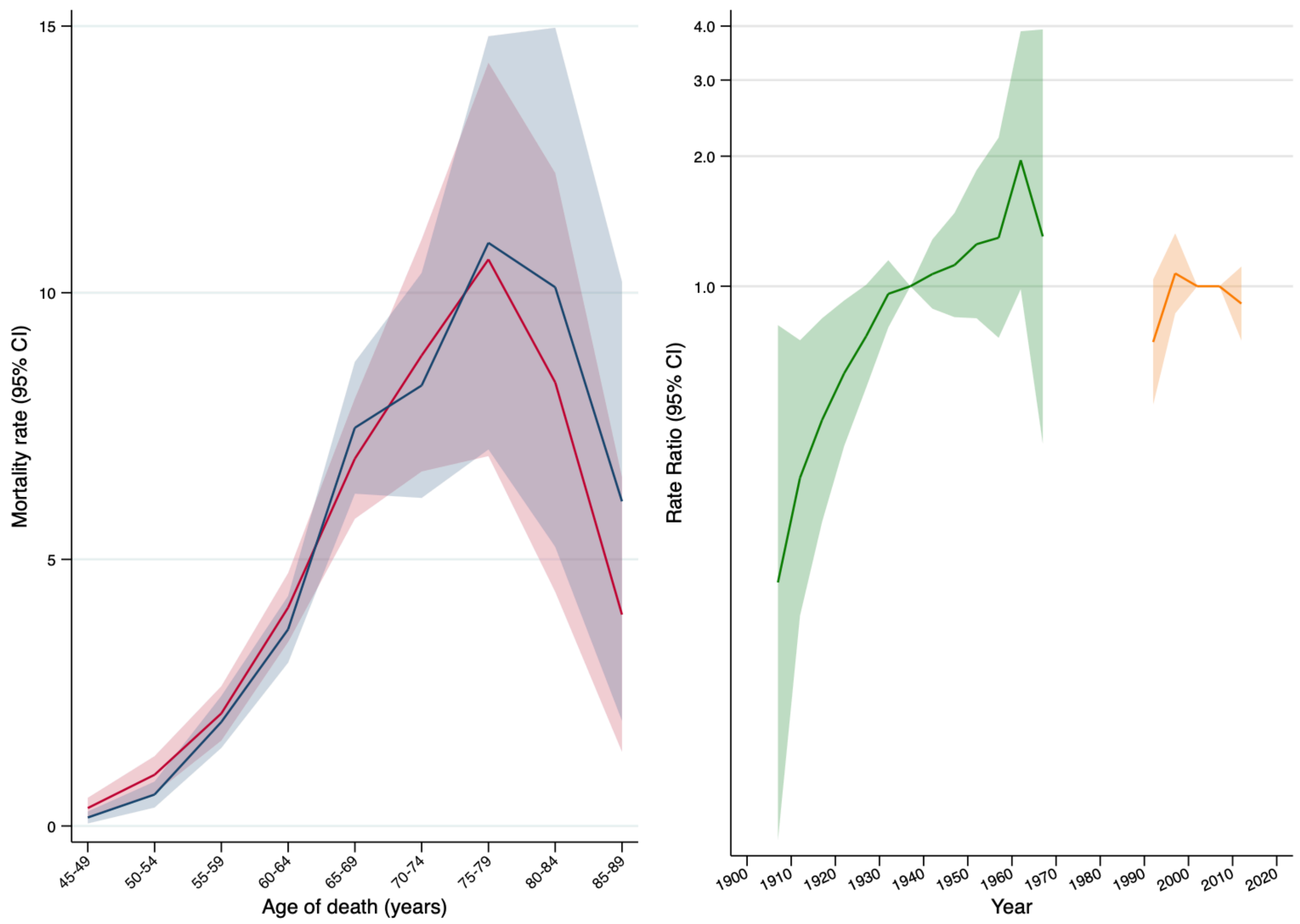

Figure S4. Age, period, and cohort effects for sCJD mortality rates: APC modeling using factor variables.
The left part of the graph shows sCJD age-specific mortality rates (per 1,000,000 person-years; shaded area, 95% confidence intervals) in men (blue) and women (red). The right part of the graph shows relative risks (shaded area, 95% confidence intervals) for periods (orange; reference, 2007) and cohorts (green; reference, 1937). Periods of death 2002-2006 and 2007-2011 are constrained to be equal (Table S5).

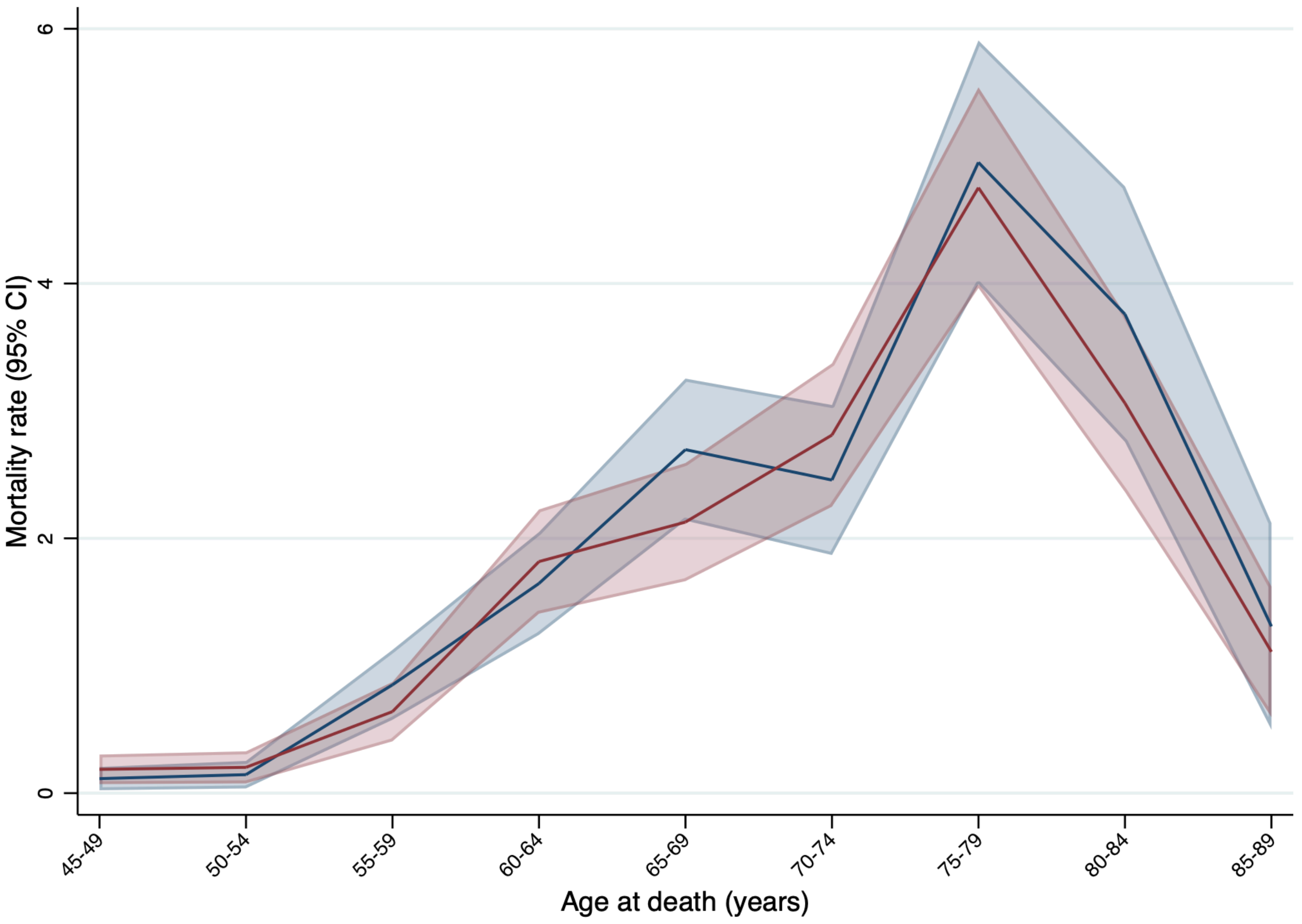

Figure S5. Age-specific mortality rates of sCJD with the M1 strain by sex (France, 1992-2016).
Age-specific mortality rates of sCJD with the M1 strain per 1,000,000 person-years in men (blue) and in women (red) with their 95% confidence intervals (Table S6).

Supplementary References

1. EuroCJD. EU case definition. https://www.eurocjd.ed.ac.uk/node/833.
2. McCormack JE, Baybutt HN, Everington D, Will RG, Ironside JW, Manson JC. PRNP contains both intronic and upstream regulatory regions that may influence susceptibility to Creutzfeldt-Jakob Disease. Gene. 2002;288(1-2):139-46.
3. Alperovitch A, Zerr I, Pocchiari M, et al. Codon 129 prion protein genotype and sporadic Creutzfeldt-Jakob disease. Lancet. 1999;353(9165):1673-4. doi:10.1016/s0140-6736(99)01342-2
4. Parchi P, Giese A, Capellari S, et al. Classification of sporadic Creutzfeldt-Jakob disease based on molecular and phenotypic analysis of 300 subjects. Annals of neurology. 1999;46(2):224-33.
5. Prusiner SB. Prions. Proc Natl Acad Sci U S A. 1998;95(23):13363-83. doi:10.1073/pnas.95.23.13363
6. Moda F, Suardi S, Di Fede G, et al. MM2-thalamic Creutzfeldt-Jakob disease: neuropathological, biochemical and transmission studies identify a distinctive prion strain. Brain pathology (Zurich, Switzerland). 2012;22(5):662-9. doi:10.1111/j.1750-3639.2012.00572.x
